# Effects of estrogen and raloxifene on synaptic density in the hippocampal CA1 region of ovariectomized rats

**DOI:** 10.1016/j.clinsp.2023.100312

**Published:** 2023-11-27

**Authors:** Glaucia Mara MenezesdaSilva, Eduardo Carvalho de Arruda Veiga, Manuel Jesus Simões, Ricardo Santos Simões, Marcos Eiji Shiroma, Maria Cândida Pinheiro Baracat, Givanna Santos Cavalcanti, Jose Maria Soares Junior, Edmund Chada Baracat

**Affiliations:** a Disciplina de Ginecologia, Departamento de Obstetrícia e Ginecologia, Hospital das Clínicas, Faculdade de Medicina da Universidade de São Paulo (HCFMUSP), São Paulo, SP, Brazil; b Disciplina de Histologia e Biologia Celular do Departamento de Morfologia da Escola Paulista de Medicina, Universidade Federal de São Paulo (UNIFESP), São Paulo, Brazil

**Keywords:** Estrogen, Hormone therapy, Raloxifene, Hippocampus, CA1 region, Ovariectomy

## Abstract

**Introduction:**

The CA1 region of the hippocampus has an important role in learning and memory. It has been shown that estrogen deficiency may reduce the synaptic density in the region and that hormone replacement therapy may attenuate the reduction.

**Objectives:**

This study aimed to evaluate the effects of estrogen and raloxifene on the synaptic density profile in the CA1 region of the hippocampus in ovariectomized rats.

**Methods:**

Sixty ovariectomized three-month-old virgin rats were randomized into six groups (n = 10). Treatments started either three days (early treatment) or sixty days (late treatment) after ovariectomy. The groups received propylene glycol vehicle (0.5 mL/animal/day), equine conjugated estrogens (50 μg/animal/day), or raloxifene (3 mg/kg/day) either early or late after ovariectomy. The drugs were administered orally by gavage for 30 days. At the end of the treatments, the animals were anesthetized and transcardially perfused with ether and saline solution. The brains were removed and prepared for analysis under transmission electron microscopy and later fixed.

**Results:**

Results showed a significant increase in the synaptic density profile of the hippocampal CA1 region in both the early estrogen (0.534 ± 0.026 µ/m^2^) and the early raloxifene (0.437 ± 0.012 µ/m^2^) treatment groups compared to the early or late vehicle-treated control groups (0.338 ± 0.038 µ/m^2^ and 0.277 ± 0.015 µ/m^2^ respectively).

**Conclusions:**

The present data suggest that the raloxifene effect may be lower than that of estrogen, even early or late treatment, on synaptic density in the hippocampus.

## Highlights

• Raloxifene effect on the CA1 hippocampus synaptic density.
• Differences between early and late hormonal treatment on CA1 hippocampus synaptic density.
• Estrogen action on the rat synaptic density is higher than one of raloxifene.

## Introduction

Estrogen receptors are widely expressed in the brain, mainly in the hippocampal region, which is associated with memory and learning. A previous study showed that, in postmenopausal women, estrogen treatment improved cognition.^1^ Other studies suggested that postmenopausal estrogen therapy played an important role in maintaining memory or preventing memory loss.^2^ Moreover, hormone replacement therapy seems to delay the onset of clinical symptoms of some neurological diseases.^3^ A growing body of evidence suggests that estrogen may affect the neurons and the synapses.^4^^,^^5^

Raloxifene is a Selective Estrogen Receptor Modulator (SERM) that can work both as an agonist and an antagonist of the estrogen receptor in different tissues.^6^^,^^7^ In rats, estrogen deprivation decreases the activity of the acetylcholine transferase enzyme; the deficit of this enzyme activity can be reversed by estrogen treatment.^8^, ^9^, ^10^, ^11^, ^12^, ^13^ Studies show that raloxifene is as effective as estrogen in enhancing acetylcholine transferase activity, which may improve cognition.^13^^,^^14^ The dose of raloxifene required to restore or elevate acetylcholine transferase activity is the same as that used to prevent postmenopausal osteoporosis[14] and bone loss in female rats.^15^ In cultured neurons, raloxifene acts as an antioxidant and reduces apoptosis.^16^^,^^17^

Estrogen enhances excitatory synapse formation in cortical neurons via a rapid extranuclear ER-mediated signaling mechanism. This mechanism involves the upregulation of the AMPA receptor GluR1 subunit and mediation by Akt and ERK signaling pathways through ERα for long, chronic periods.^17^^,^^18^ These molecular pathways are yet to be studied extensively for raloxifene. There are some differences in mechanism between estrogen and raloxifene that may result in divergent actions regarding a specific tissue.^19^, ^20^

Raloxifene's participation in acetylcholine transferase activity is well known, but its effects in a short-term or long-term treatment on synaptic density have not been fully established, especially in the hippocampus, an important region involved in memory and learning.^19^ Raloxifene is assumed to have poor penetration of the blood-brain barrier due to its molecular structure. There is some clinical evidence that raloxifene has protective action; however, this action may occur through the estrogen receptor.^20^ Therefore, using an animal model for such an evaluation is relevant.

The CA1 region in the hippocampus is considered the major outlet pathway connecting the hippocampus to the neocortex, and it is a region rich in synapses and estrogen receptors.^21^ The present study aimed to assess the effects of estrogen and raloxifene on the synaptic density profile in the CA1 region of the hippocampus in ovariectomized rats in both early (30 days) and late (60 days) administration. Secondly the hormonal effect on the uterus was evaluated through the weighting of the organ.^22^

### Materials and methods

It was an experimental and randomized animal model study. Sixty 3-month-old adult virgin albino female rats, sourced from the EPM 1-Wistar (IRB number 0326/07) colony and weighing approximately 200 grams each, were obtained from the Experimental Models Development Center (CEDEME), Federal University of São Paulo (UNIFESP). Animal experiments were conducted following the UK Animal (Scientific Procedures) Act, of 1986.

During the two-week adaptation period at the Discipline of Histology and Cell Biology, vaginal smears were collected daily. The animals underwent ovariectomy under anesthesia using ketamine (50 mg/kg) and xylazine (10 mg/kg).^23^

After the surgical procedure, the animals were randomly divided into six groups of 15 animals each: EContr ‒ Early control group, which received propylene glycol (0.5 mL/animal/day);^24^ LContr ‒ Late control group, which received propylene glycol (0.5 mL/animal/day); EEstr ‒ Early treatment group, which received an equine conjugated estrogen (50 µg/Kg/day) treatment;[25] LEstr ‒ Late treatment group, which received an equine conjugated estrogen (50 µg/Kg/day) treatment;[25] ERLX ‒ Early treatment group, which received raloxifene (3 mg/Kg/day) treatment; and LRLX ‒ Late treatment group, which received raloxifene (3 mg/Kg/day) treatment.^26^ The administration of both treatments (estrogen and raloxifene) was carried out daily by gavage.

Three groups received the drugs 3 days after ovariectomy (early treatment group) and the other three groups received the drugs 60 days after ovariectomy (late treatment group). Two animals died during the experiment: EContr (n = 1) and LContr (n = 1).

The equine conjugated estrogen (CE; Wyeth, Philadelphia, PA) used consisted of estrone (50%), equilin (25%), and dihydroequilin (15%), plus minor amounts of 17α-estradiol and 17α-dihydroequilin. The applied dose was used to cause vaginal and endometrial proliferation in previous studies.^26^ The drugs were prepared in propylene glycol, and 0.5 mL of the drug suspension was administered for 60 days by gavage with a metal probe standardized by the Department of Pharmacology, Federal University of São Paulo. At the end of the treatment, the animals were anesthetized with ketamine and xylazine.^27^, ^28^, ^29^ Subsequently, the animals were weighed and the thoracic cavity of the animals was opened with a scalpel, allowing visualization of the beating heart. The animals were transcardially perfused through a saline infusion pump with 600 U of heparin (150 mL) at 36°C.^28^^,^^29^ Next, a median longitudinal incision was made on the head, and the subcutaneous tissue and the skin were removed to visualize the skullcap.

The right hemisphere was prepared for electron microscopy. After its dissection, it was immediately dipped in a toluidine blue solution for 2 to 3 min. Next, a magnifying glass (20 ×  magnification) and a stereotactic lens were used to identify the CA1 region in the hippocampus, from which 1-mm fragments of the stratum radiatum were removed with an ophthalmic scalpel. The stratum pyramidale is a cell-dense region in the hippocampus; therefore, it was used as a reference for localizing the stratum radiatum in the hippocampal CA1 region. Also, the uterus was removed and weighed.^22^ The fragments were processed for transmission electron microscopy at the Electron Microscopy Center, Federal University of São Paulo. The dissected tissue was placed in a 0.2 M sodium cacodylate buffer, Ph 7.4, and stored overnight at 4°C. The next day, the tissue was washed five times in a sodium cacodylate solution, with a 10-minute interval between each wash. It was then postfixed in 1% osmium tetroxide solution in a 0.2 M cacodylate buffer, pH 7.4, and kept for 1h at room temperature. The tissue was washed twice with water and immediately immersed in a 0.5% aqueous solution of uranyl acetate plus sucrose for 30 min at room temperature.

**Fig. 1:**
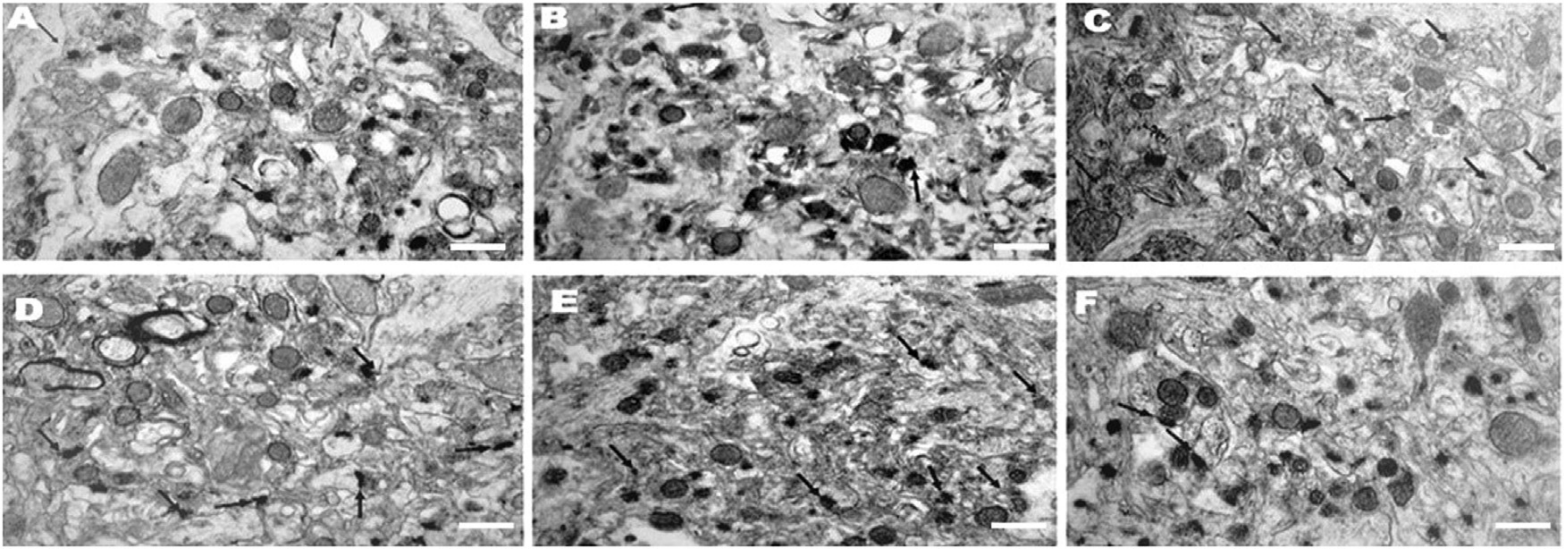
Electron micrographs of a part of the hippocampal CA1 region of ovariectomized rats in different treatment groups: (A) EControl: group of rats that received propylene glycol early; (B) LControl: group of rats that received propylene glycol late; (C) EEstr: group of rats that received estrogen early; LEstr: group of rats that received estrogen late; (D) ERLX: group of rats that received raloxifene early; (F) LRLX: group of rats that received raloxifene late. The arrows point to synapses. Scale bars equal 300 nm.

Afterward, two more washes were performed with water. The tissue was then dehydrated by incubating in 90% and in 100% ethanol for 30 min each time and the procedure was followed by two 20-min washes with propylene oxide. Inclusion was carried out with epoxy resin (Araldite) by allowing the material to spin for 1h at room temperature in a 2:1 mixture of propylene oxide and resin. The material was then placed in a 1:1 mixture of propylene oxide and resin and kept in a constant spinning motion overnight. The next day, the material was placed in pure resin under vacuum for 4h. The embedding itself was carried out in the same inclusion medium (resin). The material was kept in an oven at 60°C for 72h for polymerization. The end of the block containing the material was trimmed in the shape of a pyramid under a binocular microscope. The trimmed blocks were sectioned using an ultramicrotome (Reichert FCS Ultracut S) with a thickness between 300 nm and 600 nm. Since the right hemisphere had been previously stained with toluidine blue, the sections were examined under a light microscope to identify the regions of interest. After reconstruction, the blocks were sectioned with an ultramicrotome (Reichert FCS Ultracut S) to obtain ultrafine sections with a thickness between 75 nm and 85 nm. The sections were placed on a 200-mesh copper screen (Fig. 1).

The screen was counterstained using a saturated aqueous uranyl acetate solution for 8 min and then a lead citrate solution for 4 min. The screens were analyzed under a JEOL electronic microscope, model JEM-1200 EXII, at 80 kV. Five different areas within the same section were imaged at 5000 ×  magnification and then printed on a Kodabrome Print F3 18 × 24 paper (2.5-fold enlarged), yielding a final 12500 × magnification.

### Morphometry

To determine the synaptic density (number of synapses/µm^2^ of tissue), the authors counted the number of synapses in five random fields photographed in the hippocampal CA1 region of each animal and divided the number by the total area of the tissue. The number of synapses was counted by analyzing each photograph (12500 ×  magnification) with the aid of a stereoscopic magnifying glass with a 4 ×  magnification, resulting in a final 50000 × magnification. Each enlarged photograph was on a size 18 × 24 cm (432 cm^2^) piece of paper. Since the pictures were taken randomly, there were few areas with no synapses. These (vessels, cell nuclei, etc.) were excluded from the analysis. Also, the brain area captured in each photo was calculated.

The authors used an indirect manual counting technique based on geometrical assumptions. The principles that underlie an indirect technique for determining the number objects in a volume tissue, NV, are based on the relationship between the number of profiles of objects per unit area of the sections, QA (total number synapses/photo), the caliper height of the objects in a direction normal to the plane of the section, H (rate of synapses height/photo), and the thickness of the sections (h, 75 nm): NV = QA /(H+h). The number of synapses (N), defined as having both a postsynaptic density and at least vesicles in the presynaptic terminal no more than 0.2 μm from the synaptic cleft, was counted. In the present analysis, when a single presynaptic terminal was associated with more than one postsynaptic density on the same postsynaptic element, it was counted as a single synapse. If, however, a presynaptic terminal clearly formed synapses with more than one postsynaptic element, then this was counted as more than one synapse.^25^ Counting of synaptic densities was performed by one of us (I.S.) ‒ who, at the time of the evaluation, was blinded to the animal's experimental condition.

### Statistical analysis

The sample size was calculated using the Sample Size Calculator through https://wnarifin.github.io/ssc/ssanimal.html. The minimal total number of animals was 18 for The ANOVA design is one-way ANOVA, applied for group comparison and power calculation of 90% based on the previous study on the synaptic density of CA1 hippocampus.^25^

The statistical methods used were the following: summary measures based on a normal distribution (median, mean, and standard error), coefficients of asymmetry, and kurtosis; the Kolmogorov-Smirnov test for estimating Gaussian distributions; and Levine's test for characterizing the homogeneity of variances. The bar graph represents the mean synaptic density in the hippocampal CA1 region of the adult ovariectomized rats.

The analysis of variance (ANOVA) was used for comparing the groups, and it was followed by the post hoc Tukey test when significant differences between the groups were found in order to establish which groups differed significantly. Analyses were carried out using Sigma Stat 2.0 (Fandel Scientific, New York, USA) software. A p-value less than 0.05 was considered statistically significant.

## Results

The qualitative morphological analysis using transmission electron microscopy revealed significant qualitative changes in the axonal and dendritic synaptic profiles of the hippocampal CA1 region in the different treatment groups (Fig. 1 A‒F). The values of the mean and standard error were expressed as percentages, and the synapses visualized in the electron micrographs were counted and also expressed as a percentage. The results showed that the early estrogen treatment significantly increased (50.9% ± 4.1%) the synaptic density in the hippocampal CA1 region when compared to the results of the early control group (p < 0.01). Similarly, the late estrogen treatment led to a significant increase (21.3% ± 5.9%, p < 0.01) in the synaptic density profile of the hippocampal CA1 region when compared to the results of the late control group. Moreover, the early and late treatments with raloxifene caused a statistically significant increase (32.1% ± 6.3% and 15.8% ± 2.1% respectively) in the synaptic density of the hippocampal CA1 region when compared to the early and the late control groups respectively (Fig. 1, p < 0.03). The early raloxifene treatment was less effective (32.1% ± 6.3%) than the early estrogen treatment (50.9% ± 4.1%, p < 0.01). No significant difference was found between the late estrogen and the late raloxifene treatments.

Among the present results, synaptic density, per µm^2^ (mean, standard deviation, median) of the CA1 region of the hippocampus of ovariectomized rats (radiated stratum) in the various groups studied is described as follows: no EContr group (n = 14) The authors obtained a mean of 0.338 with a standard deviation of 0.038 and a median of 0.343. In the LContr group (n = 14) the results were a mean of 0.277 with a standard deviation of 0.015 and a median of 0.274, and in the EEstr group (n = 15) a mean of 0.534 with a standard deviation of 0.026 and a median of 0.532, followed by the group LEstr (n = 15) with mean of 0.355 with a standard deviation of 0.014 and median of 0.351. The last two groups obtained the following results in the ELRX group (n = 15) with a mean of 0.437 with a standard deviation of 0.012 and a median of 0.441 in the LRLX group (n = 15) and a mean of 0.340 with a standard deviation of 0.011 and a median of 0.339.

**Fig. 2:**
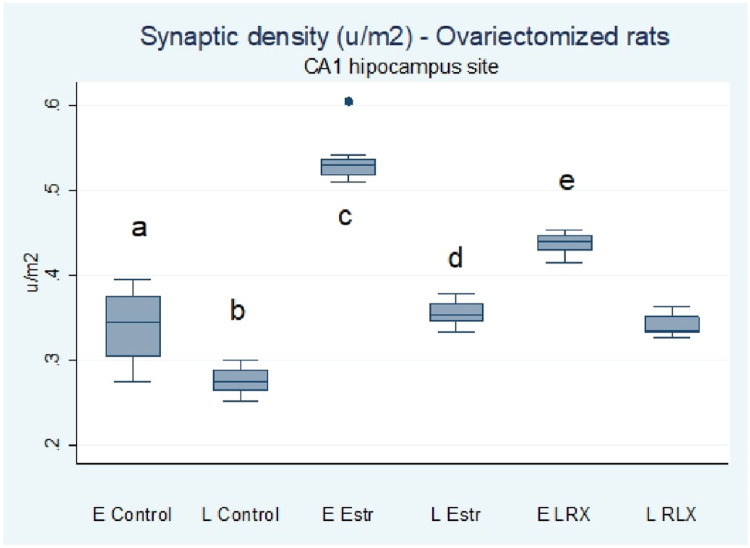
Graphical representation of synaptic density in the hippocampal CA1 region of the ovariectomized rats. EControl, group of rats that received propylene glycol early; LControl, group of rats that received propylene glycol late; EEstr, group of rats that received estrogen early; LEstr, group of rats that received estrogen late; ERLX, group of rats that received raloxifene early; LRLX, group of rats that received raloxifene late. Population distribution was analyzed by the Kolmogorov-Smirnov test. One-way ANOVA and the post hoc Tuckey test were performed for comparing the groups. ^a^ p < 0.001 compared to LContr, EEstr, and ERLX; ^b^ p < 0.001 compared to EEstr, LEstr, ERLX, and LRLX; ^c^ p < 0.001 compared to LEstr, ERLX, LRLX; ^d^ p < 0.001 compared to ERLX; ^e^ p < compared to LRLX. F value is 254,5.

The quantitative data are represented in Fig. 2. One-way ANOVA and the post hoc Tuckey test were performed for group comparison. The F value was 254.5. The data were expressed as synapsis per µm^2^. The early estrogen treatment (EEstr, 5.37 ± 0.22) was the most effective of all the treatments (p < 0.01) in terms of synaptic density: EContr (3.51 ± 0.53), LControl (2.89 ± 0.26), LEstr (3.74 ± 0.24), ERLX (4.47 ± 0.19) and LRLX (3.53 ± 0.34).

The synaptic density of the animals in the LContr group was the least affected. The difference was significant (p < 0.001) in comparison with all of the other groups (EContr, EEstr, LEstr. ERLX, and LRLX. The values of the ELRX group were higher than those of the early and late controls, LEstr, and LRAX (p < 0.01).

Multiple Comparison Testing (one-way ANOVA followed by the post hoc Tukey test) (Table 1) for assessing the number of transmission electron microscopy fields without synapses showed the groups did not differ statistically: EEstr (n = 6), LEstr (n = 6), EContr (n = 3), LContr (n = 4), ELRX (n = 7), and LRLX (n = 5). The photos with no synapses were discarded and not included.

**Table 1:** Multiple comparison testing (ANOVA one way followed by posthoc of Tukey).

**Comparation**	**Differences of Averages**	**p**
EContr vs. LContr	0.0605	0.001
EEstr vs. EContr	0.197	0.001
EEstr vs. LContr	0.257	0.001
EEstr vs. LEstr	0.180	0.001
EEstr vs. ERLX	0.0969	0.001
EEstr vs. LRLX	0.194	0.001
LEstr vs. EContr	0.0169	NS
LEstr vs. LContr	0.0774	0.001
LEstr vs. ERLX	0.0829	NS
LEstr vs. LRLX	0.0146	0.001
ERLX vs. EContr	0.0998	0.001
ERLX vs. LContr	0.160	0.001
ERLX vs. LRLX	0.0974	0.001
LRLX vs. EContr	0.00234	NS
LRLX vs. LContr	0.0628	0.001

NS, Not Significant.

**Fig. 3:**
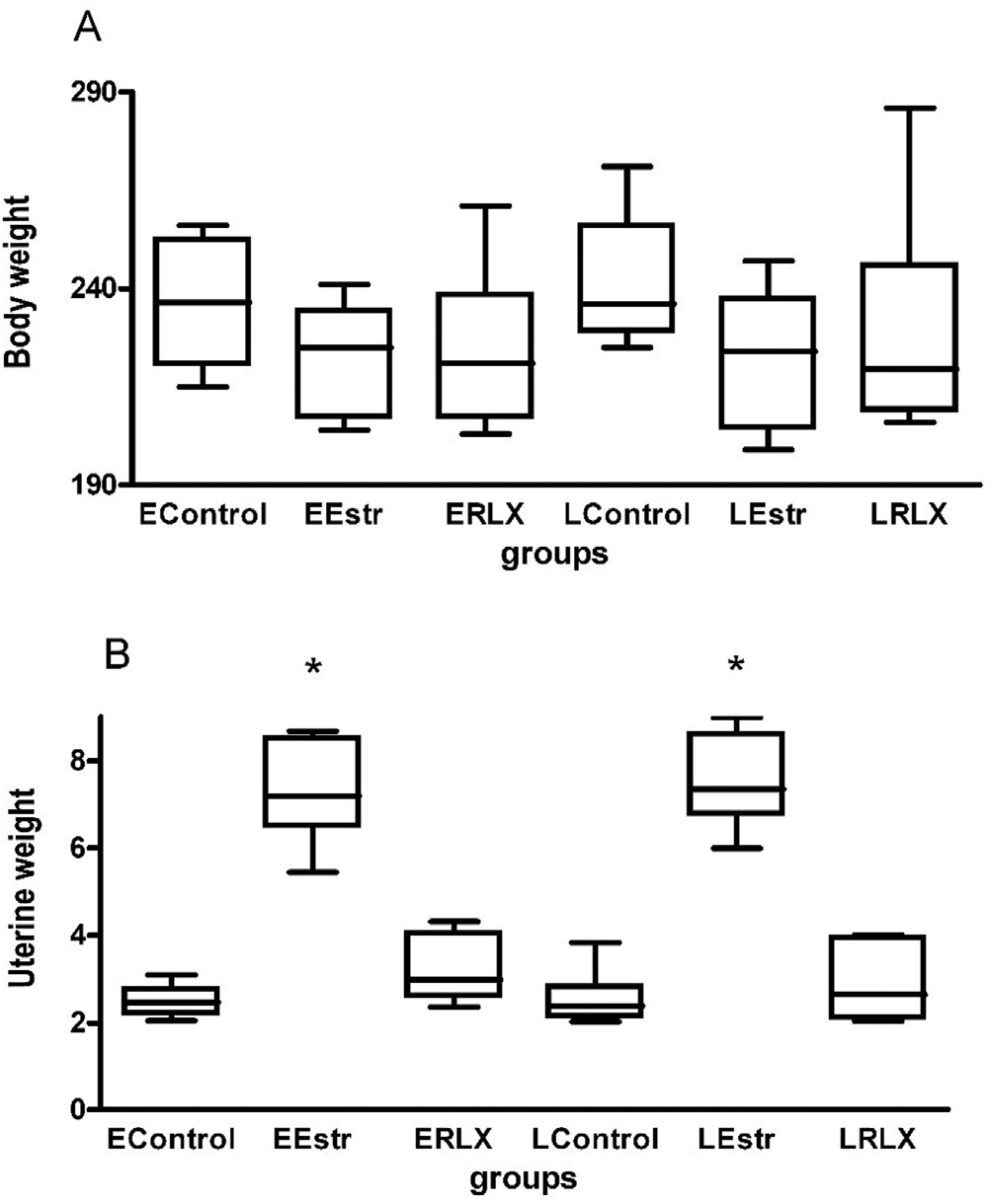
Graphical representation: (A) Body weight values. The groups are not different (Anova test); (B) Uterine weight values. *p < 0.001 compared to other groups. There is no difference between EEstr and LEstr. The ANOVA test was applied, and it was followed by the Tukey test.

The present results demonstrate that, when administered initially, estrogen increases synaptic density. In addition, compared to raloxifene, estrogen produced a higher increase in synaptic density, both in the early and late treatment groups. Fig. 3A shows that the rat body weights in the different treatment groups were similar (p = 0.074). Both the early and the late estrogen groups exhibited the highest values of uterine weight (Fig. 3B). Furthermore, synaptic density was found not to correlate with rat weight.

## Discussion

The main result of the present work was the improvement by estrogen in synaptic density in both early and late groups, and when compared to raloxifene there was an improvement in synaptic density in the groups of animals that were treated with estrogen. In fact, this study showed that, in ovariectomized rats, early as against late administration of estrogen determined an increase in synapses. The onset of treatment may be important for neurons. Also, the raloxifene treatment exhibited a similar behavior.

Estrogen regulates the anatomy and the connections of the hippocampus with other associated structures. Studies suggest that the ability of estrogen to improve the performance of spatial memory tasks in ovariectomized rodents depends on age, estrogen dose, and duration of hypoestrogenism before the initiation of estrogen treatments.^30^ This is because there is an opportunity window for estrogen to act on neurons, and cells do not respond to estrogen after a long period of low levels of this hormone in adult animals.^31^ In the study, estrogen treatment improved the spatial memory in 3-month-old ovariectomized rats when the treatment was initiated early rather than very late after ovariectomy.^19^ Estrogen levels fall after menopause, a period when many central nervous system activities deteriorate, particularly hippocampal functions, such as memory, attention, cognition, and automatic control.^30^ In the rat brain, the hippocampal CA1 region is involved in regulating similar activities.^31^ Besides, estrogen might enhance neuronal excitability during the proestrus phase relative to the metestrus phase of the rat estrous cycle and potentiate neuronal excitability by activating the NMDA receptors and affecting the GABAergic metabolism in the female rodent.^32^ These studies may explain the effect of estrogen on synaptic density.

One of the challenging questions investigators have been trying to address is the exact role of SERMs in the central nervous system. Raloxifene may protect against epilepsy, cerebral ischemia, brain damage, aging, and Alzheimer's disease through various signaling pathways, act as an antioxidant and antiapoptotic, and enhance the expression of cytoskeleton proteins.^33^ The estrogen's ability to improve memory tasks may be explained by the increases in synaptic density in the CA1 region prompted by the early treatments with estrogen or raloxifene; estrogen, however, produced better results than raloxifene. Moreover, raloxifene interacts with several cell membrane receptors, such as Insulin-like Growth Factor 1 Receptor (IGF-1R) and Epidermal Growth Factor Receptor (EGFR), and stimulates the effector molecules, including Src, Phosphatidylinositol 3-Kinase (PI3K), serine/threonine-protein kinase (Akt), and Mitogen-aActivated Protein Kinase (MAPK), leading to uncontrolled cell proliferation and cancer.^34^ Perhaps these interactions underlie the differences between the estrogen and the raloxifene treatments.

These results are consistent with those of previous studies, mainly those focused on early treatment after ovariectomy.^5^ They show that the group receiving estrogen initially was statistically different from all other groups in the study, that is, the EEstr group produced the highest synaptic density. They also show that the ELRX group had a higher synaptic density than the controls and the late estrogen and late raloxifene groups. However, when the authors compared EEstr and ELRX, it was found that the group initially treated with estrogen had a higher synaptic density than the group initially treated with raloxifene. These results agree with the literature.^35^

Among the strengths of the study is that the synaptic density in the hippocampal CA1 region of the ovariectomized rats increased significantly when estrogen or raloxifene treatment was initiated early. Furthermore, results show that the animals that received estrogen early after ovariectomy had a greater increase in synaptic density than the animals that received estrogen late after ovariectomy. Additionally, the present results show that both raloxifene and estrogen were effective in preventing a decrease in synaptic density in the hippocampal region when administered early. Still, there was a slight protection in late administration. Dendritic spine density in CA1 pyramidal cells is sensitive to naturally occurring estrogen fluctuations in young animals and to experimentally induced estrogen depletion and replacement. Recent evidence suggests that estrogens mediate these morphological changes through *N-*methyl-d-aspartate (NMDA) receptors.^36^ Also, estrogen regulates protein synthesis and actin polymerization in hippocampal neurons through stimulation of mTOR activity.^37^

The present data has some clinical implications based on the estrogen treatment in postmenopausal women might have beneficial cognitive effects.^38^ The clear demonstration of such clinical behavioral effects, however, has not always been easily made.^39^ Therefore this experimental data may support this estrogen effect. The great question of literature is the impact of raloxifene on the central nervous system, memory, and cognition in postmenopausal women. There is some evidence of positive effects on working memory.^40^ The authors chose the CA1 hypothalamus due to it is adequate for evaluating synapses. Also, this area in rats is related to memory in the present data, raloxifene increased the number of synapsis in the CA1 hypothalamus area, but this action is lower than one of estrogen. However, the present data may support the clinical findings of raloxifene on postmenopausal women.^40^

Among the limitations of the studies, the authors understand that raloxifene action was limited owing to the fact that this study was based solely on histological aspects, It is necessary in future studies to carry out another randomized experimental study in rats to obtain a greater understanding of the mechanisms of raloxifene and estrogen in the synaptic density of the hippocampus of these animals. There is a need for further studies of functional gain, such as memory and learning, of changes in the neurotransmitter level, which is indicative of increased neuronal activity, and of the molecular mechanisms involved in estrogen- and raloxifene-mediated increases in synaptic density.

## Conclusion

The present data suggest that the raloxifene effect may be lower than that of estrogen, even early or late treatment, on synaptic density in the hippocampus. Further studies are required to understand the mechanisms underlying the mode of action of raloxifene on the central nervous system.

## Conflicts of interest

The authors declare no conflicts of interest.
